# Mechanical Power in Prone Position Intubated Patients with COVID-19-Related ARDS: A Cohort Study

**DOI:** 10.1155/2023/6604313

**Published:** 2023-03-01

**Authors:** Roberto Stalla Alves da Fonseca, Viviane Martins Correa Boniatti, Michelle Carneiro Teixeira, Alessandra Preisig Werlang, Francielle Martins, Pedro Henrique Rigotti Soares, Leonardo da Silva Marques, Wagner Luis Nedel

**Affiliations:** ^1^Intensive Care Unit, Hospital Nossa Senhora da Conceição, Porto Alegre, Brazil; ^2^Programa de Pós-Graduação em Cardiologia, Universidade Federal do Rio Grande do Sul, Porto Alegre, Brazil; ^3^Programa de Pós-Graduação em Bioquímica, Universidade Federal do Rio Grande do Sul, Porto Alegre, Brazil; ^4^Brazilian Research in Intensive Care Network (BRICNet), São Paulo, Brazil

## Abstract

**Background:**

Respiratory monitoring of mechanical ventilation (MV) is relevant and challenging in COVID-19. Mechanical power (MP) is a novel and promising monitoring tool in acute distress respiratory syndrome (ARDS), representing the amount of energy transferred from the ventilator to the patient. It encompasses several setting parameters and patient-dependent variables that could cause lung injury. MP can therefore be an additional tool in the assessment of these patients.

**Objective:**

This study aims to evaluate respiratory monitoring through MP and its relationship with mortality in patients with COVID-19-related ARDS (CARDS) under mechanical ventilation (MV) and prone position (PP) strategies.

**Methods:**

Retrospective, unicentric, and cohort studies. We included patients with CARDS under invasive MV and PP strategies. Information regarding MP, ventilation, and gas exchange was collected at 3 moments: (1) prior to the first PP, (2) during the first PP, and (3) during the last PP. We tested the relationship between MP and VR with in-hospital mortality.

**Results:**

We included 91 patients. There was a statistically significant difference in MP measurements between survivors and nonsurvivors only in the last prone position (*p* < 0.001). This is due to the significant increase in MP measurements in nonsurvivors (difference from the baseline: 3.63 J/min; 95% CI: 0.31 to 6.94), which was not observed in the group that survived (difference from the baseline: 0.02 J/min; 95% CI: −2.66 to 2.70). In multivariate analysis, MP (*p*=0.009) was associated with hospital death when corrected for confounder variables (SAPS 3 score, mechanical ventilation time, age, and number of prone sessions).

**Conclusions:**

MP is an independent predictor of mortality in PP patients with CARDS.

## 1. Introduction

The prone position (PP) is a cornerstone of invasive ventilatory supply in moderate to severe acute distress respiratory syndrome (ARDS) and has been a current recommendation in COVID-19-related ARDS (CARDS) [1, 2]. This maneuver is associated with a reduction in mortality in patients with severe ARDS compared to the supine position, as well as an improvement in oxygenation measured by the change in the PaO_2_/FiO_2_ ratio [2].

The patient's response to PP is defined primarily as an improvement in oxygenation, especially through the PaO_2_/FiO_2_ ratio. It should be noted that the main reason for reducing mortality in PP is less overdistension in nondependent lung regions and less cyclical opening and closing in dependent lung regions [1, 3]. Determining the response to PP using a single oxygenation index can limit the possible benefits of this therapy. Possibly, the concomitant evaluation of changes in other variables of respiratory monitoring may provide relevant information for better ventilatory support [4]. Due to its combined effects of recruiting vertebral parts of the lung, making the distribution of ventilation more homogeneous, and reducing intracycle recruitment/derecruitment, PP dampens lung stress and strain resulting from mechanical ventilation (MV) [3], decreasing the risk of ventilator-induced lung injury (VILI) [5].

More recently, the degree of VILI has been associated with the energy load transferred from the mechanical ventilator to the patient's respiratory system, converting the mechanical stimulus into intracellular biochemical and molecular signals. Some studies have shown that manipulation of individual ventilatory parameters does not add benefit in terms of reducing VILI if it does not cause a concomitant reduction in dynamic strain and energy/power load [6, 7]. Mechanical power (MP) represents the amount of energy transferred from the ventilator to the patient per unit of time. This index includes several setting parameters and patient-dependent variables that could cause VILI in a single measurement [8]. Recent studies have suggested that monitoring ventilatory mechanics performed by driving pressure (ΔP) and MP may be more appropriate when a customized ventilation strategy is aimed [6, 7]. PP have a well-known effect on new open pulmonary units and improve the mechanical characteristics of already opened units that reach a more favorable position on the volume-pressure curve [1], associated with a more homogeneous ventilation distribution [3]. These phenomena may theoretically have an impact on the energy load delivered to the lungs [8], reflecting on MP. In a previous study, PP associated with a PEEP titration strategy minimized the parameters associated with VILI, such as MP [9].

As a result of this theoretical effect of PP on a lower and more homogeneous distribution of energy in the lung parenchyma [8], the objective of our study is to evaluate MP as a monitoring tool for ventilatory mechanics, the longitudinal trend throughout hospitalization, and prone-induced changes in intubated patients with moderate-to-severe CARDS.

## 2. Materials and Methods

### 2.1. Study Design

In this retrospective study, we collected data from patients admitted between 5th May and 9th September 2020 to a tertiary intensive care unit (ICU) in Brazil. The local ethics committee approved the study (Grupo Hospitalar Conceição Ethics Comitee-Plataforma Brasil number CAAE 51855421600005530—approval date: October 20^th^, 2021). The informed consent requirement was waived due to the retrospective and noninterventionist nature of the study. This study was performed in accordance with the ethical standards of the responsible committee on human experimentation and with the Helsinki Declaration of 1975. The inclusion criteria for the analysis were patients with laboratory-confirmed SARS-CoV-2 infection (i.e., a positive result of a real-time reverse transcriptase—polymerase chain reaction assay of nasal and pharyngeal swabs), who had moderate to severe ARDS according to the Berlin criteria [10] and who required intervention therapy with mechanical ventilation and prone position. The application of prone position therapy was carried out according to the Proseva trial criteria [2], with prone sessions of at least 16 hours. MV management was carried out with strategies that limit tidal volumes (4–8 ml/kg of predicted body weight) and inspiratory pressures (plateau pressure <30 cm H_2_O) [11]. PEEP values are titrated with the aim, primarily, of a plateau pressure <30 cm H_2_O and, ideally, also a Δ*P* < 15 cm H_2_O. Recruitment maneuver was not performed in each session of prone position. In the population included in this study, prone sessions interrupted by hemodynamic instability were not identified, and patients with severe hemodynamic instability were not included. All patients had a continuous measurement of invasive blood pressure using an arterial catheter. Patients were preferentially ventilated in volume-controlled ventilation (VCV), and all patients received continuous infusions of neuromuscular blocker drugs during the protocol. The infusion rate of drugs for sedation and analgesia in these patients was titrated through bispectral index (BIS) monitoring.

Multiple information was collected from the electronic medical record, including admission data (demographic and anthropometric data and comorbidities) and data on clinical evolution during the ICU stay period, including ventilatory settings parameters (i. e. positive end expiratory pressure (PEEP), tidal volume (Vt), respiratory rate (RR), FiO_2_), ventilatory monitoring (i.e. peak pressure (Ppeak), plateau pressure (Pplat), ΔP, respiratory system compliance (Crs), minute ventilation (VE)) and monitoring pulmonary gas exchange (PaCO_2_ and PaO_2_/FiO_2_ ratio, and ventilatory ratio [VR]). Vt was reported in ml/kg of predicted body weight (PBW). ΔP was defined as the difference between Pplat and PEEP. Static compliance (Cst) was calculated as Vt/(Pplat − PEEP). VR was calculated from the following equation: VR = [VE (ml/min) × PaCO_2_ (mmHg)]/(PBW × 100 × 37.5). MP was expressed in J/min and calculated as previously proposed by Chiumello et al. [12] from the following equations: MP (J/min) = 0.098 × RR × Vt × Ppeak − 0.5 × (Pplat − PEEP) when in volume-controlled ventilation and MP (J/min) = 0.098 × RR × Vt × (ΔPinsp + PEEP) in pressure-controlled ventilation. The ventilatory setting parameters and respiratory variables monitoring data were collected at three moments: (1) before the first prone position, (2) during the first prone position (after at least 6 hours), and (3) during the last prone position. The primary outcome was hospital mortality.

### 2.2. Statistical Analysis

Continuous variables that were normally distributed were presented as mean and standard deviations (SD). Nonnormally distributed continuous variables were represented by medians and interquartile ranges (IQR). Categorical data are presented as absolute numbers (*n*) and percentages (%). The Mann–Whitney test was used to compare continuous nonparametric variables between study groups. Pearson's chi-square test was used for categorical variables. A generalized estimating equation was performed to explore the interaction between the different variables measured (MP, VR, PaO_2_/FiO_2_ ratio, driving pressure, PEEP, and tidal volume) and the effect of time of mensuration (immediately before the first prone position, during the first session of the prone position, and in the last prone position), as well as its interaction with survival at ICU discharge. This modeling also allows us to evaluate the interaction in both time and survival status with each variable (interaction survival × time) and also to evaluate the impact of MP in survival status when corrected for confounding variables (SAPS III, number of days on MV, number of prone maneuvers, and age). We performed an exploratory analysis evaluating the difference between MPs at the three time points: MP in the first prone minus MP preprone (delta [∆]MP 1), MP in the last prone minus MP preprone (∆ MP 2), and the MP in the last prone minus MP in the first prone (∆ MP 3). Statistical significance was defined as *p* < 0.05. Statistical analysis was performed with SPSS software 21.0 (SPSS, IBM—Chicago, Illinois, USA) and jamovi 2.3.18.

## 3. Results

During the study period, data were collected from a total of 91 COVID-19 ARDS intubated patients in a prone position. The ICU and the hospital mortality rate were 49% (*n* = 45). Most of the patients were men (63.7%); the mean age was 60.2 ± 12.8 years; and the median body mass index was 30 (26.8–34.6) kg/m^2^. The mean simplified acute physiology score (SAPS) 3 on admission to the ICU was 68.6 ± 15.5 points, and the mean sequential acute organ failure assessment (SOFA) score was 7 ± 2.3 points. The median number of prone maneuvers in the overall cohort was 2 sessions (IQR 1–4).  Table 1 summarizes the clinical characteristics of the patients and their clinical outcomes.

**Figure 1:**
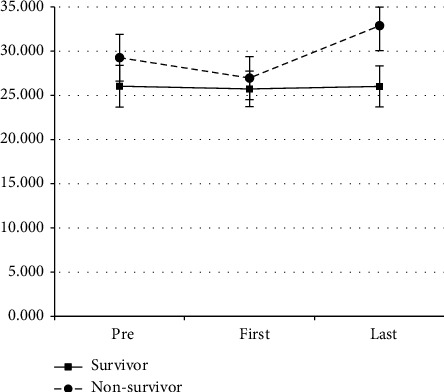
MP and VR measurements in the three prone position moments: preprone, first prone, and last prone maneuver. MP, mechanical power (in J/min).

Figure 1 shows a comparison of MP at three moments of assessment stratified by survival status. Survivors had nonstatistically significant lower values of MP in the preprone interval when compared with nonsurvivors: 25.5 J/min (21.5–29.9) versus 28.3 J/min (28.3–36.2), *p*=0.07. During the first prone, there was no difference in MP when comparing survivors and nonsurvivors: 24.5 J/min (22.1–28.4) vs. 25.7 J/min (21.7–30), *p*=0.546, respectively. During the last prone session, survivors had lower MP measurements when compared with nonsurvivors: 26.1 J/min (21.6–30) vs. 32.8 J/min (26.1–38.2), *p* < 0.001. Some baseline characteristics were higher in nonsurvivors, including mean age (65.6 ± 11.8 vs. 55.0 ± 11.6; *p* < 0.001) and SAPS 3 score (73.7 ± 17.1 vs. 63.8 ± 12.1; *p*=0.002), as well as prevalence of COPD (22.2% vs. 2.2%; *p*=0.03). The number of prone sessions was also significantly higher in nonsurvivors [2.0 (1.0–4.0) vs. 3.0 (2.0–5.5); *p*=0.03]. The need for renal replacement therapy was significantly higher in nonsurvivors (57.8% vs. 26.1%; *p*=0.004). There was no statistically significant difference in the incidence of venous thromboembolism.

There was no difference between survivors and nonsurvivors in ∆ MP 1 (mean difference 2.8 J/min, 95% CI −0.33 to 5.93; *p*=0.08) or in ∆ MP 2 (mean difference −3.3 J/min, 95% CI −7.84 to 1.25; *p*=0.152). Survivors, however, had a statistically significant lower ∆ MP 3 when compared with nonsurvivors (mean difference −8.73 J/min, 95% CI −13.92 to −3.53; *p*=0.001).

The ventilatory setting and monitoring parameter data collected in the preprone position, during the first prone position, and in the last prone position are presented in Table 2. There was a statistically significant difference in MP measurements between survivors and nonsurvivors only in the last prone position (*p* < 0.001). This is due to the significant increase in nonsurvivors in the last prone position (difference from baseline: 3.63 J/min; 95% CI: 0.31 to 6.94), which was not observed in the group that survived (difference from baseline: 0.02 J/min; 95% CI: −2.66 to 2.70). There was a statistically significant interaction between survival status and time of measurement (pre, first, and last prone positions) in the parameters MP (*p*=0.009), VR (*p*=0.009), RR (*p* < 0.001), and PaO_2_/FiO_2_ ratio (*p*=0.001) in a model adjusted for potential confounders. There was an interaction between Δ*P* and PEEP measurements only with respect to the moment of measurement and not with the survival status.

## 4. Discussion

In this study, we propose that, in patients with CARDS submitted to PP, the variability of MP presents different dynamics in survivors and in nonsurvivors, and these results persist even after adjustment for potentially confounding variables. The hospital mortality rate in this cohort was consistent with that presented in other studies for the same patients with moderate-to-severe CARDS under MV [11, 12].

The association between MP and mortality in acute respiratory failure due to COVID-19 was also observed in a secondary analysis of the PRoVENT-COVID study, where MP was independently associated with mortality at 28 days [13]. The mean MP in our study is comparable to another study in the field [14], and, despite the lack of a universally accepted MP threshold to guide the proper use of MV, lower levels than those found in our study are already consistently associated with increased mortality [15, 16]. Furthermore, MP values tend to be higher in CARDS than in ARDS due to other etiologies [17], possibly because patients with CARDS request high ventilatory demands to maintain acceptable PaCO_2_ and pH, which require higher Vt and RR. The physiological impact generated by PP may suggest an association between the maneuver and its impact on MP. Its effect on the recruitment of vertebral parts of the lung, making the ventilation distribution more homogeneous [3], may dampen lung stress and strain due to an increase in the surface of the lung that is capable of accommodating energy transfer [8]. However, few data explored the effect of PP on MP in patients with CARDS, with variability trends similar to those of our study [18].

We also observed a difference in variability between the three moments of measurement in the PaO_2_/FiO_2_ ratio in survivors and nonsurvivors. There was also a trend of longitudinal increase in the MP during the PP strategy when comparing the values obtained during the first and last prone positions in relation to the baseline value, with a greater increase detected in nonsurvivors. This finding may suggest that not only the level of MP should have an impact on the outcome but also that the duration of parenchymal exposure to it can cause additional lung injury [19]. Although the results of our work may demonstrate that the magnitude of the improvement in oxygenation due to PP may be related to better results, we consider that the association between the improvement in the PaO_2_/FiO_2_ ratio and improved outcomes is an issue that needs to be clarified in further studies. In ARDS patients, while Gattinoni et al. observed that the “PaO_2_ responders” (those who increased PaO_2_/FiO_2_ by 20 mmHg) had an outcome similar to that of nonresponders [20], Scaramuzzo et al. demonstrate that a sustained improvement in oxygenation of PP after resupination would be associated with improved clinical outcomes [5]. Our study points to an association between high MP values and hospital mortality in severe forms of COVID-19. However, patients with COVID-19 have a high incidence of long-term pulmonary alterations in survivors, with a relevant impact on the quality of life of this population [21]. In further studies, it will be relevant to evaluate the impact of different ventilatory mechanical variables on the long-term outcomes of this population.

There are several limitations to our study. Due to the observational nature of the study, therapeutic assistance cannot be standardized. The patients were preferentially ventilated in VCV mode. All patients received continuous infusions of neuromuscular blockade drugs, and the infusions of sedative and analgesic drugs were titrated by BIS monitoring. There has not been a uniform PEEP titration strategy during the prone maneuver, despite the fact that PEEP has variable responses in patients with COVID-19. In a previous study, the shunt fraction, alveolar dead space, and ventilation/perfusion matching were not affected by PEEP [22]. However, a potential impact of PEEP titration on MP cannot be assessed, as suggested in a previous study [9]. Our PEEP settings, however, are similar to other work in this field [23, 24]. The observed PBW values for Vt/kg were slightly higher than the recommended 6 ml/kg [25]. However, we do not believe that this fact has an important influence on the results, considering that (1) there were no statistically significant differences between survivors and nonsurvivors; (2) other LPV measurements were respected, including an average Δ*P* < 15 cm H_2_O in both groups; and (3) as already described above, patients with CARDS tend to experience relatively good lung compliance and, according to some references, larger tidal volumes (7-8 ml/kg of PBW) without worsening the risk of VILI [26].

## 5. Conclusions

MP appears to comprise adequate respiratory monitoring to provide more personalized and adaptive ventilatory support. More prospective trials are needed to test whether this strategy is capable of improving the outcome of mortality.

## Figures and Tables

**Table 1:** Baseline characteristics and outcomes of included patients.

Variables	Overall population (*n* = 91)	Survivors (*n* = 46)	Nonsurvivors (*n* = 45)	*p* value
*Clinical characteristics*				
Age (years)^*∗*^	60.2 ± 12.8	55.0 ± 11.6	65.6 ± 11.8	<0.001
Gender (male), *n* (%)	58.0 (63.7)	29.0 (63.0)	29.0 (63.4)	0.88
BMI (kg/m^2^)^†^	30.0 (26.8–34.6)	30.9 (27.8–35.1)	29.1 (25.7–32.6)	0.09
Asthma, *n* (%)	2.0 (2.2)	2.0 (4.3)	0.0 (0.0)	0.15
COPD, *n* (%)	11.0 (12.1)	1.0 (2.2)	10.0 (22.2)	0.003
Systemic arterial hypertension, *n* (%)	62.0 (68.1)	31.0 (67.4)	31.0 (68.9)	0.87
Diabetes mellitus, *n* (%)	33.0 (36.3)	15.0 (32.6)	18.0 (40.0)	0.46
Chronic kidney disease, *n* (%)	7.0 (7.7)	2.0 (4.3)	5.0 (11.1)	0.22
SAPS 3^*∗*^	68.6 ± 15.5	63.8 ± 12.1	73.7 ± 17.1	0.002
SOFA score	7 ± 2.7	7 ± 3	7 ± 2.1	0.73

*Outcomes*				
Number of prone maneuvers	2.0 (1.0–4.0)	2.0 (1.0–4.0)	3.0 (2.0–5.5)	0.03
VET, *n* (%)	17.0 (18.7)	10.0 (21.7)	7.0 (15.6)	0.62
RRT, *n* (%)	38.0 (41.8)	12.0 (26.1)	26.0 (57.8)	0.004
ICU LOS (days)^†^	23.0 (12.0–34.0)	25.5 (11.7–37.0)	20.0 (12.0–31.0)	0.24
Hospital LOS (days)^†^	29.0 (19.0–46.0)	35.0 (21.7–59.5)	26.0 (16.0–33.0)	0.004

BMI: body mass index; COPD: chronic obstructive pulmonary disease; ICU: intensive care unit; LOS: length of stay; RRT: renal replacement therapy; SAPS: simplified acute physiology score; SOFA: sequential organ failure assessment; VET: venous thromboembolism. ^*∗*^Mean ± standard deviation; ^†^median (P25–P75).

**Table 2:** Ventilatory settings and monitoring parameters during pre-, first-, and last-prone sessions.

Variables^*∗*^	Survivors	Nonsurvivors	*p* value	Effects (*p* value)^*∗*^		
				Survival	Time	Survival × time
Mechanical power (J/min)				0.004	0.004	0.009
Preprone	26.0 ± 2.34^a^	29.2 ± 2.64^a^	0.073			
First prone	25.7 ± 2.0^a^	26.9 ± 2.4^a^	0.445			
Last prone	26.0 ± 2.3^a^	32.9 ± 2.8^b^	<0.001			
Ventilatory ratio				<0.001	<0.001	0.009
Preprone	2.0 ± 0.14^a^	2.4 ± 0.2^a^	<0.001			
First prone	2.1 ± 0.16^ab^	2.5 ± 0.17^a^	<0.001			
Last prone	2.2 ± 0.16^b^	3.0 ± 0.24^b^	<0.001			
PaO_2_/FiO_2_ ratio				0.001	<0.001	0.001
Preprone	115.0 ± 4.0^a^	117.0 ± 3.0^a^	0.144			
First prone	270.0 ± 20.0^b^	202.0 ± 11.0^b^	0.003			
Last prone	283.0 ± 20.0^b^	181.0 ± 11.0^b^	<0.001			
Driving pressure (cm H_2_O)				0.570	<0.001	0.467
Preprone	13.6 ± 0.45^b^	13.6 ± 0.51^b^	0.971			
First prone	11.7 ± 0.5^a^	12.3 ± 0.5^a^	0.360			
Last prone	11.6 ± 0.54^a^	12.5 ± 0.78^ab^	0.325			
PEEP (cm H_2_O)				0.794	0.008	0.089
Preprone	12.0 ± 0.26^a^	12.0 ± 0.4^b^	0.929			
First prone	12.0 ± 0.24^a^	11.4 ± 0.39^a^	0.185			
Last prone	12.3 ± 0.33^a^	12.6 ± 0.42^b^	0.643			
Tidal volume (ml/kg PBW)				0.842	0.435	0.397
Preprone	6.6 ± 0.13^a^	6.8 ± 0.15^a^	0.329			
First prone	6.6 ± 0.12^a^	6.7 ± 0.15^a^	0.797			
Last prone	6.5 ± 0.11^a^	6.7 ± 0.14^a^	0.378			
Respiratory rate (mpm)				0.025	<0.001	0.482
Preprone	23.7 ± 3.6^a^	25.2 ± 3.7^a^	0.045			
First prone	25.2 ± 3.4^a^	26.2 ± 3^a^	0.136			
Last prone	25.8 ± 3.5^a^	27.4 ± 3.7^a^	0.04			

PaO_2_ = arterial oxygen pressure; FiO_2_ = inspired oxygen; PEEP = end-expiratory positive pressure.^*∗*^Mean ± standard deviation. ^*∗*^*p* values adjusted by SAPS III, number of days on MV, and number of prone maneuvers and age; ^ab^equal letters do not differ by the least significant difference (LSD) test at 5% significance.

## Data Availability

The data supporting the findings of the current study are available from the corresponding author upon request.

## Abbreviations

ARDS: Acute distress respiratory syndrome
BIS: Bispectral index
CARDS:COVID-related ARDSCrs: COVID-related ARDSCrs:Respiratory system compliance
DP: Driving pressure
ICU: Intensive care unit
MP: Mechanical power
MV: Mechanical ventilation
PBW: Predicted body weight
PEEP: Positive end-expiratory pressure
PP: Prone position
Ppeak: Peak pressure
Pplat: Plateau pressure
RR: Respiratory rate
VCV: Volume-controlled ventilation
VILI: Ventilator-induced lung injury
VE: Minute ventilation
VR: Ventilatory ratio
Vt: Tidal volume.

## References

[1] Guérin C., Albert R. K., Beitler J., Gattinoni L., Jaber S., Marini J. J. (2020). Prone position in ARDS patients: why, when, how and for whom. *Intensive Care Medicine*.

[2] Guérin C., Reignier J., Richard J.-C., Beuret P., Gacouin A., Boulain T. (2013). Prone positioning in severe acute respiratory distress syndrome. *New England Journal of Medicine*.

[3] Papazian L., Munshi L., Guérin C. (2022). Prone position in mechanically ventilated patients. *Intensive Care Medicine*.

[4] Chiumello D., Bonifazi M., Pozzi T., Formenti P., Papa G. F. S., Zuanetti G. (2021). Positive end-expiratory pressure in COVID-19 acute respiratory distress syndrome: the heterogeneous effects. *Critical Care*.

[5] Scaramuzzo G., Gamberini L., Tonetti T., Zani G., Ottaviani I., Mazzoli C. A. (2021). Sustained oxygenation improvement after first prone positioning is associated with liberation from mechanical ventilation and mortality in critically ill COVID-19 patients: a cohort study. *Annals of Intensive Care*.

[6] Tonetti T., Vasques F., Rapetti F., Maiolo G., Collino F., Romitti F. (2017). Driving pressure and mechanical power: new targets for VILI prevention. *Annals of Translational Medicine*.

[7] Gattinoni L., Tonetti T., Cressoni M., Cadringher P., Herrmann P., Moerer O. (2016). Ventilator-related causes of lung injury: the mechanical power. *Intensive Care Medicine*.

[8] Silva P. L., Ball L., Rocco P. R. M., Pelosi P. (2019). Power to mechanical power to minimize ventilator-induced lung injury?. *Intensive Care Medicine Experimental*.

[9] Boesing C., Graf P. T., Schmitt F., Thiel M., Pelosi P., Rocco P. R. M. (2022). Effects of different positive end-expiratory pressure titration strategies during prone positioning in patients with acute respiratory distress syndrome: a prospective interventional study. *Critical Care*.

[10] Ranieri V. M., Rubenfeld G. D., Thompson B. T., Ferguson N. D., Caldwell E., Fan E. (2012). Acute respiratory distress syndrome: the Berlin Definition. *JAMA*.

[11] Weiss C. H., McSparron J. I., Chatterjee R. S., Herman D., Fan E., Wilson K. C. (2017). Summary for clinicians: mechanical ventilation in adult patients with acute respiratory distress syndrome clinical practice guideline. *Ann Am Thorac Soc*.

[12] Chiumello D., Gotti M., Guanziroli M., Formenti P., Umbrello M., Pasticci I. (2020). Bedside calculation of mechanical power during volume- and pressure-controlled mechanical ventilation. *Critical Care*.

[13] Schuijt M. T. U., Schultz M. J., Paulus F., Serpa Neto A., van Akkeren J. P., Algera A. G. (2021). Association of intensity of ventilation with 28-day mortality in COVID-19 patients with acute respiratory failure: insights from the PRoVENT-COVID study. *Critical Care*.

[14] Schmidt M., Hajage D., Demoule A., Pham T., Combes A., Dres M. (2021). Clinical characteristics and day-90 outcomes of 4244 critically ill adults with COVID-19: a prospective cohort study. *Intensive Care Medicine*.

[15] Serpa Neto A., Deliberato R. O., Johnson A. E. W., Bos L. D., Amorim P., Pereira S. M. (2018). Mechanical power of ventilation is associated with mortality in critically ill patients: an analysis of patients in two observational cohorts. *Intensive Care Medicine*.

[16] Cressoni M., Gotti M., Chiurazzi C., Massari D., Algieri I., Amini M. (2016). Mechanical power and development of ventilator-induced lung injury. *Anesthesiology*.

[17] Becker A., Seiler F., Muellenbach R. M., Danziger G., Kamphorst M., Lotz C. (2021). Pulmonary hemodynamics and ventilation in patients with COVID-19-related respiratory failure and ARDS. *Journal of Intensive Care Medicine*.

[18] Laghlam D., Charpentier J., Hamou Z. A., Nguyen L. S., Pene F., Cariou A. (2022). Effects of prone positioning on respiratory mechanics and oxygenation in critically ill patients with COVID-19 requiring venovenous extracorporeal membrane oxygenation. *Frontiers of Medicine*.

[19] Silva P. L., Pelosi P., Rocco P. R. M. (2020). Understanding the mysteries of mechanical power. *Anesthesiology*.

[20] Gattinoni L., Vagginelli F., Carlesso E., Taccone P., Conte V., Chiumello D. (2003). Decrease in PaCO2 with prone position is predictive of improved outcome in acute respiratory distress syndrome. *Critical Care Medicine*.

[21] Scaramuzzo G., Ronzoni L., Campo G., Priani P., Arena C., la Rosa R. (2022). Long-term dyspnea, regional ventilation distribution and peripheral lung function in COVID-19 survivors: a 1 year follow up study. *BMC Pulmonary Medicine*.

[22] Scaramuzzo G., Karbing D. S., Fogagnolo A., Mauri T., Spinelli E., Mari M. (2022). Heterogeneity of ventilation/perfusion mismatch at different levels of PEEP and in mechanical phenotypes of COVID-19 ARDS. *Respiratory Care*.

[23] Ferrando C., Suarez-Sipmann F., Mellado-Artigas R., Hernández M., Gea A., Arruti E. (2020). Clinical features, ventilatory management, and outcome of ARDS caused by COVID-19 are similar to other causes of ARDS. *Intensive Care Medicine*.

[24] Gleissman H., Forsgren A., Andersson E., Lindqvist E., Lipka Falck A., Cronhjort M. (2021). Prone positioning in mechanically ventilated patients with severe acute respiratory distress syndrome and coronavirus disease 2019. *Acta Anaesthesiologica Scandinavica*.

[25] Brower R. G., Matthay M. A., Morris A., Schoenfeld D., Taylor Thompson B. (2000). Ventilation with lower tidal volumes as compared with traditional tidal volumes for acute lung injury and the acute respiratory distress syndrome. *New England Journal of Medicine*.

[26] Marini J. J., Gattinoni L. (2020). Management of COVID-19 respiratory distress. *JAMA*.

